# A domain sequence approach to pangenomics: applications to *Escherichia coli*

**DOI:** 10.12688/f1000research.1-19.v2

**Published:** 2013-05-29

**Authors:** Lars-Gustav Snipen, David W Ussery

**Affiliations:** 1 Department of Chemistry, Biotechnology and Food Sciences, Norwegian University of Life Sciences, Ås, Norway; 2 Centre for Biological Sequence Analysis, Technical University of Denmark, Lyngby, Denmark

## Abstract

The study of microbial pangenomes relies on the computation of gene families, i.e. the clustering of coding sequences into groups of essentially similar genes. There is no standard approach to obtain such gene families. Ideally, the gene family computations should be robust against errors in the annotation of genes in various genomes. In an attempt to achieve this robustness, we propose to cluster sequences by their domain sequence, i.e. the ordered sequence of domains in their protein sequence. In a study of 347 genomes from *Escherichia coli* we find on average around 4500 proteins having hits in Pfam-A in every genome, clustering into around 2500 distinct domain sequence families in each genome. Across all genomes we find a total of 5724 such families. A binomial mixture model approach indicates this is around 95% of all domain sequences we would expect to see in *E. coli* in the future. A Heaps law analysis indicates the population of domain sequences is larger, but this analysis is also very sensitive to smaller changes in the computation procedure. The resolution between strains is good despite the coarse grouping obtained by domain sequence families. Clustering sequences by their ordered domain content give us domain sequence families, who are robust to errors in the gene prediction step. The computational load of the procedure scales linearly with the number of genomes, which is needed for the future explosion in the number of re-sequenced strains. The use of domain sequence families for a functional classification of strains clearly has some potential to be explored.

## Introduction

Microbial pangenomics has attracted interest over recent years, stimulated by the availability of sequence data from whole-genome re-sequencing projects ^1– 7^. The pangenome of a prokaryotic species is the collection of gene families for the entire species, as opposed to a single genome, which is the set of genes in a functional organism. The pangenome diversity can be huge, which is also reflected in the span of phenotypes. An example of this is found in the model organism *Escherichia coli* ^8, 9^. Currently there are more than a thousand *E. coli* genomic projects listed ^10^ and this number will grow in the near future, along with genomes for many other bacteria; it is reasonable to assume that pangenomics will attract more attention.

The fundamental step in any pangenome analysis is the computation of gene families. Several approaches to computing gene families have been used in previous pangenome analyses ^1, 11, 12^, but this part of the analysis has received little attention. A pangenome analysis typically involves the estimation of the size of the core and the pangenome, measured by the number of gene families, and several methods have been proposed for doing this ^1, 11, 13, 14^. The core is the set of gene families present in all genomes of the population. The remaining gene families are more or less abundant among the genomes. The sample pangenome size is the total number of gene families found in the currently available genomes, while the population pangenome size is the number of gene families we expect to see if every single strain was sequenced. It is the latter which is of scientific interest, but it must be estimated from the former. A fraction of the gene families will be found only in a small number of genomes, and those observed in only a single genome are called ORFans.

The first step of a gene family computation is to obtain a list of protein coding genes for each genome under study. Completed genomes will have a set of annotated genes, but the quality of these annotations may vary between projects. A pangenome analysis will often include draft genomes as well, where annotations are lacking. For these reasons it is convenient to start the analysis by a gene-finding step, treating all genomes the same way, and minimizing variability due to different annotation procedures. Even if prokaryotic genes are in most cases simpler to detect than eukaryotic counterparts, there are still problematic cases ^15^. In case of the draft genomes, where the genome sequence is spread out on a (large) number of contigs, the gene-finding problem is even more difficult. Ideally, we would like to compute gene families in a way that minimizes the effect of various gene finding algorithms.

The second step is to group proteins into gene families. A gene family is basically the set of orthologs and in-paralogs collected from the various genomes. The most common approach so far is based on all-against-all alignments to compute some kind of similarity/distance between all proteins, and then finally cluster these. This approach poses some problems. First, the all-against-all approach is not computationally feasible in the long run since the number of genomes grows rapidly. Second, the clustering procedures always involve some granularity threshold implicitly defining the size/number of gene families. Some kind of thresholding seems impossible to avoid, but it would be desirable to allow it some variability over gene families, since some gene families are expected to be more conserved than others. Finally, false predicted ‘genes’ will not align well to any other, and produce singelton clusters adding, sometimes dramatically, to the total number of gene families found.

When grouping protein coding genes, we find it natural to consider the presence of common protein domains. Domains are closely linked to gene function ^16^. Previous work has demonstrated that the functional repertoire of a genome can be predicted by the knowledge of domain content ^17, 18^. In addition, focusing on the combination of domains in each protein, instead of the frequency of each domain separately, we come even closer to the protein function ^19, 20^. A grouping of proteins by function rather than by evolutionary distance seems like a good alternative, since pangenomics is in many cases about the study of functional diversity.

The Pfam-A database ^21^ is a comprehensive collection of domains, curated and of high quality. Each entry is characterized by a profile hidden Markov model, and until recently it has been quite time consuming to search against such a database. However, with the launching of the HMMER3 software ^22, 23^, it is now possible to search with all proteins within a genome against the entire Pfam-A database in reasonable time (few minutes), even on a laptop. The idea of using domain information in comparative genomics is not new, and Yang *et al.* used genome-wide domain frequencies to reconstruct phylogenetic trees ^24^. Their study considered diverse genomes from both prokaryotes, eukaryotes and archaea, quite opposite of pangenomics, where we focus only on genomes from the same species. An argument for using domains was that structure is more conserved than sequence, and that domain information provides a view into ancient history ^24^. In this perspective one may question the use of domain information for pangenomics studies, since they may provide too low resolution.

In this paper we consider domain sequence families defined by the domain sequence of each protein as an alternative to gene families for pangenome studies. The domain sequence is the ordered sequence of non-overlapping domains along the protein. Clusters computed in this way will be coarse compared to previously used gene families, but are closely linked to gene function. We describe the procedure of computing such gene families, and consider the effect of gene prediction and of the use of draft genomes in the analysis. We use this approach in a pangenome study of the model organism *Escherichia coli*, the prokaryotic species with the largest set of available genomes.

## Materials and methods

The methods we have used for gene prediction and database scanning are all standard and free software, see below. We acknowledge that the gene finders have tuning possibilities, and that slightly different results may be obtained by using this. We have used only default settings, or settings suggested in accompanying manuals, in order to make the approach as universal as possible. The data are from the model organism *Escherichia coli* only, and many of the more specific results cannot be extended to other species. However, the procedures are applicable to all prokaryotes where we have genomes from many strains available.

### Data

Genome sequences for *Escherichia coli* were downloaded from NCBI ^25^ on June 15 2012. At that time, there were 56 completed and 291 draft genomes available as contigs.

### Gene finders

In a pangenome study involving draft genomes some automated gene finding is needed. For each genome we predicted genes using the three popular gene finders Prodigal ^26^, GeneMark ^27^ and Glimmer ^28^.

The Prodigal gene finder (v2.60) was run by the command line

**prodigal -i gnom.fsa -o pred.txt -q**

where **gnom.fsa** was a FASTA-formatted input file with the genome sequences, and **pred.txt** was the output file.

The GeneMark predictions were obtained by first training a Hidden Markov Model (HMM) using GeneMark.S (v4.6b):

**gmsn.pl –combine –clean –gm gnom.fsa**

which produced a model file **GeneMark.hmm.combined.mod**. Next, this was used to predict genes by **GeneMark.hmm**, prokaryote version 2.6:

**gmhmmp -m GeneMark.hmm.combined.mod -o pred.txt gnom.fsa**

The Glimmer predictions were obtained by the tigr-glimmer wrapper software for linux. First, the long.orfs software was used to extract training sequences from the genome sequences:

**tigr-glimmer long-orfs -n -l -t 1.15 -g 30 seq.fsa coord.txt tigr-glimmer extract -t seq.fsa coord.txt >> train.fsa**

where **seq.fsa** was the FASTA file with a single genome sequence. This was repeated by looping through all genome sequences, producing the file **train.fsa** with training sequence data. Next, an Interpolated Context Model (ICM) was trained:

**tigr-glimmer build-icm -r glim.icm < train.fsa**

where **glim.icm** was the model file output. This was finally used to make gene predictions

**tigr-glimmer glimmer3 -o50 -g110 -t30 –linear –extend gnom.fsa glim.icm pred.txt**

In all cases the gene predictions were stored as a table with one row for each predicted gene, and with columns GenomeSequence (name of genome sequence where it is found), Strand (1 or -1), Left (smallest coordinate), Right (largest coordinate) and Partial (logical indicating if the gene runs over the start/end of the genome sequence). This format makes comparison of gene predictions straightforward.

### Effect of gene prediction

We wanted to examine the effect of various gene finders on the list of proteins obtained from each genome. For this study we only focused on the 54 completed genomes with annotated proteins in the RefSeq database ^29^. Given the curation of the RefSeq database, and the fact that *E. coli* is the most well studied prokaryotic genome, we expect these RefSeq annotations to be as close to the truth as one can expect for any prokaryotic organism. Thus, by using the three gene finders described above we obtained four sets of proteins for each of the 54 genomes. We compared these sets using the Jaccard distance defined as

$$J\left(S_{a},S_{b}\right)=1-\frac{\left|S_{a}\cap S_{b}\right|}{\left|S_{a}\cup S_{b}\right|}(1)$$

where *S _a_* and *S _b_* are two sets of proteins and |.| indicates cardinality (number of elements in the set). A distance of 0.0 means the two sets *S _a_* and *S _b_* are identical, while the maximum distance of 1.0 means they are non-overlapping. In this comparison, two proteins were considered equal only if they were exactly identical (identical amino acid sequence). After extraction of domain sequences from each protein (see below) we again computed Jaccard distances. This time two proteins were considered equal if they had identical domain sequence.

### Final gene prediction

For the sake of completeness all genomes, both complete and draft, were subjected to the same analysis. Since we subsequently filtered all results through the Pfam-A database (see below), we were less concerned about false positive gene predictions at this step. To obtain a comprehensive gene-list, we ran all three gene-finders on each genome, and compiled the union of the results into one long list of ORFs for each genome. In the cases where the same stop-codon had alternative starts, we always chose the longer ORF, again due to the later Pfam-A filtering. All partial ORFs (lacking start and/or stop codon) were eliminated before the analysis. We made no attempt to resolve overlaps between different ORFs. Finally, all remaining ORFs were translated to protein.

### Pfam search

FASTA-formatted files of protein sequences were queried against the Pfam-A database version 26.0 ^21^ using the HMMER 3.0 software ^22, 23^. The search was done using the command line

**hmmscan -o out.txt –cut_ga –noali –cpu 0 –domtblout res.txt Pfam-A.hmm prot.fsa**

where **res.txt** is the name of the output file, **Pfam-A.hmm** is the HMMER3 database and **prot.fsa** is the FASTA-formatted input file of protein sequences to scan. We used the option **–cut_ga** in order to invoke the curated inclusion thresholds inherent in the Pfam-A models. The iEvalue (independent E-value) was used as the criterion in the cases where we wanted to overrun these thresholds, using a stricter level of significance.

Proteins giving no hits in Pfam-A were discarded from the downstream analysis. This step presumably filtered out the majority of the false positive gene predictions from the gene-finding step. We observed that predicted proteins with Pfam-A hits did in general not overlap each other on the genomes, but no restrictions were imposed to reduce or filter out those overlaps that occurred.

### Domain sequences

For every protein sequence the significant hits were listed in their order of appearance along the protein. In the cases where two or more domains were overlapping, the one with largest E-value was eliminated, and this was repeated in a recursive procedure until no more overlaps between hits were found. There is a small number of nested domains (domains inside domains) in the Pfam database, but we chose to eliminate all occurrences of overlaps. The *domain sequence* of a protein is the ordered list of Pfam accession numbers, and multiple hits of the same domain may occur as long as they are non-overlapping. It is a high-level alternative to the amino acid sequence representation of a protein. Finally, we grouped into domain sequence families all proteins having identical domain sequence.

### Pan-matrix

The fundamental data structure for a pangenome analysis is the pan-matrix. This matrix has one row for each genome and one column for each domain sequence or gene family, and cell ( *i, j*) holds the number of copies of domain sequence family *j* in genome *j*, alternatively just 0 or 1 to indicate absence/presence, the latter being used in this paper. Let *x _j_* be the number of genomes in which family *j* is present. It is natural to view *x _j_* as a random variable, the randomness mainly due to our random selection of genomes since there are obviously many genomes we could have, but have not yet, looked into. The variable *x _j_* can take any integer value from 1 to *G*, where *G* = 347 is the number of genomes in this study.

Each row of the presence/absence pan-matrix is a coordinate vector indicating the location of the corresponding genome in a domain sequence space. This domain sequence space is closely related to a functional space in the sense that neighbors in this space are likely to display similar functions. This space is high-dimensional (many different functions), but in order to visualize the genomes relative location in this space we used a principal component decomposition to extract the most dominant directions. The genomes location along these dominant directions give us some indications of their relative location in the functional space.

### Heaps law and population closedness

Using a Heaps law type of regression model, Tettelin *et al.* made an estimate of pangenome closedness ^13^. A closed pangenome means the gene families are sampled from a stable and finite set, as opposed to an open pangenome where gene families ‘migrate’ in and out at unknown rates. If we arrange the *G* = 347 genomes in some fixed order, we can define *n _g_* as the number of new families seen in genome *g* compared to the previous *g* – 1 genomes. The Heaps law says that

$$E\left(n_{g}\right)=\beta g^{-\alpha }(2)$$

where β is some intercept and α is the parameter of interest. An α below 1 indicates the number of new families does not decrease fast enough for the pangenome to be limited, i.e. if the number of genomes *G* grows towards infinity the number of new families observed will never reach zero. Parameters can be estimated from data, by repeatedly permuting the order of the genomes and counting *n _g_* for *g* = 2, …, *G*, after each re-ordering.

### Binomial mixture model analysis

As explained above, let *x _j_* be the number of genomes in which we observe domain sequence family *j*. Let *y _g_* be the number of families found in *g* genomes (number of *x _j_*’s with value *g*). Then *y _g_* is also a random variable. The probability density of this variable, e.g. the one depicted in Figure 3, can be described by a *K* component binomial mixture model

$$f_{y}=\sum_{k=1}^{K}\pi_{k}b\left(\rho_{k}\right)$$

where *b*( *ρ _k_*) is the binomial density with probability *ρ _k_* and *π _k_* is the mixing proportion (the *π _k_*’s sum to 1.0). The left pie of Figure 7 is a visualization of a *K* =12 component model, where the size of each sector is the corresponding *π _k_* and the color of each sector indicates *ρ _k_*, for κ =1, …, 12. Summing *y _1_, y _2_, …, y _347_* we get the number of domain sequence families seen so far, i.e. the sample pangenome size. From the binomial mixture model we can also predict *y _0_*, the number of families not yet seen, and in this way we can estimate the population pangenome size ^11, 14^. The probabilities *ρ _k_* we refer to as the *selection probabilities*. A domain sequence family with selection probability 0.1 will on average be present (selected) in 1 out of 10 genomes.

Genome diversity can also be expressed as the overlap between genomes, and for this we could use the mean Jaccard distance defined above, where *S _a_* and *S _b_* are two sets of domain sequences (two genomes). Genome fluidity, introduced in ^30^, is an almost identical measure. A small Jaccard distance/genome fluidity indicates a small degree of uniqueness in each genome, i.e. large overlap. The *expected overlap* between two genomes can also be computed directly from a fitted binomial mixture model in an elegant way. The expected number of domain sequence families in a genome is

$$E_{1}=N\sum_{k=1}^{K}\rho_{\kappa }\pi_{\kappa }$$

where *N* is the population pangenome size. The expected number of domain sequences found in two independent genomes simultaneously is

$$E_{2}=N\sum_{k=1}^{K}\rho_{\kappa }^{2}\pi_{\kappa }$$

since the probability of selecting a domain sequence family twice is *ρ _k_* · *ρ _k_*, given that *ρ _k_* is its selection probability. The expected overlap between two genomes can be expressed as *E*_2_/ *E*_1_. Note that the pangenome size *N* cancels out, and the result only depends on the selection probabilities in combination with the mixing proportions. The expected overlap between 3 and more genomes can be computed along the same lines.

## Results and discussion

### Gene prediction

Before we commenced the full scale analysis, we wanted to investigate the effect of gene finding algorithms. To this end, we considered only the 54 complete *E. coli* genomes with annotations in the RefSeq database ^29^ at the time of this study. In Table 1 we show how the sets of genes differ between the official RefSeq annotation and the three most commonly used gene finders for prokaryotes. From the upper triangle of the table we see that the Jaccard distance to the RefSeq annotations is large in all cases. Even for Prodigal, which comes closest to the RefSeq annotations, the distance is 0.26. For a list of 5000 proteins, this corresponds to around 750 proteins on each list not being found in the other. Also the differences between the gene finders are large, and there is a severe uncertainty in any list of genes obtained by any single gene finder. In the lower triangle of Table 1 we see how the differences are reduced dramatically once we only consider domain sequences. This means all proteins without Pfam-A hits are discarded, and the remaining proteins are only compared by the domain sequence. The difference in start codon predictions, causing the majority of differences in the first comparison, no longer have any impact. If two proteins have the same domains, it does not matter how they compare outside these domains.

**Table 1.** Gene finding. For each of the 54 RefSeq-annotated *E. coli* genomes we compared the set of annotated genes and the set of predicted genes from Prodigal, Glimmer and GeneMark. The Jaccard distance between all pairs of sets was computed, see Method section. First, two genes were identical only if they have identical amino acid sequence. The upper triangle (above the diagonal) shows the mean Jaccard distance over the 54 genomes in this case. Next, we computed the Pfam-A domain sequence of each predicted protein, discarding all proteins without Pfam-A hits, and again computed the Jaccard distance between the sets. The lower triangle (below the diagonal) shows the mean Jaccard distance over the 54 genomes in this case.

	RefSeq	Prodigal	GeneMark	Glimmer
RefSeq		0.26	0.40	0.77
Prodigal	0.03		0.35	0.78
GeneMark	0.04	0.02		0.78
Glimmer	0.35	0.34	0.34	

In the RefSeq annotated genomes we found on average around 2436 unique domain sequence families (from 2257 to 2628). Using the union of the three gene finders, we found on average 2549, i.e. 113 domain sequence families more. A more detailed counting showed that from the RefSeq genomes around 12 families (0.5%) from each genome is not found by any of the three gene finders. On the other hand, the three gene finders find on average 125 other domain sequences families in each genome compared to the RefSeq annotations. This means that either the RefSeq annotated genomes have missed quite a number of domain-containing protein sequences, or the built-in inclusion thresholds in the Pfam-A models are too liberal, resulting in a substantial number of false positive hits. To investigate the latter, we tried out stricter significance thresholds in the HMMER3 software. Even for an E-value of *E* = 10 ^-10^ we found around 106 extra domain sequence families per genome in the union of the three gene finders compared to the RefSeq annotation. We believe this illustrates that annotated genomes still contain several ‘holes’, which is also the conclusion of others ^15^. The fact that gene finding in prokaryotes is simpler than in eukaryotes may have created an illusion that it is straightforward and nearly 100% reliable, when careful studies have shown that typical bacterial genomes have around 10% genes that are false positive, and another 10% of the proteins discovered by proteomics are missing ^31– 33^. Angiuoli *et al.* suggested an annotation procedure based on the whole-genome alignment of all genomes in a pangenome study, and found a disturbing amount of inconsistencies in public annotations ^34^. For *E. coli* less than 50% of the gene-structures were consistently annotated across 30 genomes, and the majority of the inconsistencies were due to differences in start codons positions.

### *E. coli* domain sequences

**Figure 1.**
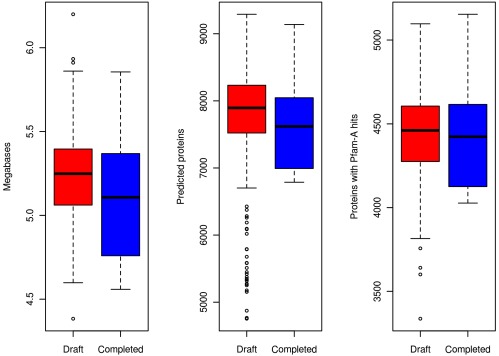
Complete and draft genomes. The box and whisker plots illustrate the differences between completed and draft genomes in this study. The left panel shows that the 56 complete genomes are somewhat smaller in size measured in megabases. This is most likely due to unresolved overlaps between the contigs in the draft genomes. The middle panel contains the number of unique genes predicted by the three gene finders after the elimination of all partial predictions (lacking start or stop codon). Notice the large number of predicted genes in virtually all cases, annotated *E. coli* genomes usually have 4500–5500 genes. Among the draft genomes some genomes have very few predicted genes, seen as circles. The rightmost panel shows the number of predicted genes with at least one Pfam-A hit. Except from four draft genomes with extremely few genes, the differences between complete and draft genomes are now ignorable.

The input to the analysis was a set of 347 genomes of *E. coli*, 56 of these were completed and 291 were available as contigs, all public data downloaded from NCBI ^25^. The left panel of Figure 1 shows the size distribution for the completed and the draft genomes. The first step was to predict genes in all genomes by three different gene finders, as explained in the Methods section. As seen in the center panel of Figure 1, this resulted in a list of roughly 7000–8000 predicted genes in each genome, which is far more than the 4500–5500 genes usually found annotated for *E. coli* genomes. This reflects the disagreement between the gene finders, and we expect a large proportion of false positives in this set. The second step was to scan all predicted proteins against the Pfam-A database. Protein sequences producing no hits in Pfam-A were discarded from the downstream analysis. Roughly 4000–5000 proteins from every genome produced significant hits in Pfam-A, see Figure 1, right panel. We also note that the difference between completed and draft genomes is more or less nonexistent after this step. This justifies the use of draft genomes in pangenome studies like this.

Considering all sequences from the 347 genomes, a total of 3679 unique Pfam-A domains/families produced significant hits, which is approximately 30% of the entire database. This produced a total of 5724 unique domain sequence families for the 347 *E. coli* genomes, with around 2500 in any single genome (2549 for the completed genomes, slightly less for the draft genomes). This is a small number compared to the number of gene families previously found for *E. coli* ^9^. The domain sequence families are expected to be fewer and larger clusters compared to the previously computed gene families. First, not all genes have domains listed in Pfam-A, and these are discarded here. This significantly shortens the list of clusters, presumably also eliminating a large majority of the false positive gene predictions. Second, some domain sequence families are large, containing proteins sharing perhaps only a single domain. In these cases a domain sequence family may contain more than one gene family.

**Figure 2.**
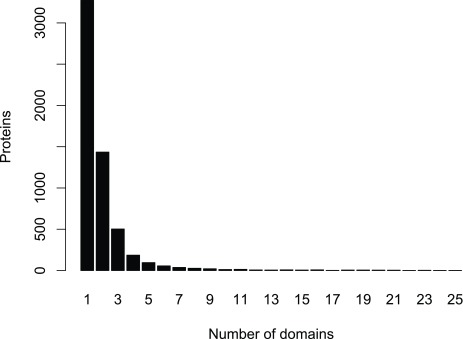
Domain sequence lengths. The bar chart shows how many domain sequences have 1 domain, 2 domains, … etc up to 25 domains. These are all non-overlapping hits in the protein. Single-domain proteins makes up more than half of the total number.

The most common domain sequence family in *E. coli* is the single-domain protein containing the major facilitator superfamily (MFS) transporter with accession PF07690.11. This is found in around 50 copies in every single genome. Other very frequently occurring single-domain proteins are the binding-protein-dependent transport system inner membrane component (PF00528.17), and the ATP-binding domain of ABC-transporters (PF00005.22). Among the multi-domain proteins the sequence PF00126.22, PF03466.15 is the most common. This is a domain sequence characteristic of HTH-type transcriptional regulators, and it occurs in more than 30 copies in each of the 347 *E. coli* genomes investigated here. Figure 2 shows that more than 3000 of the domain sequence families are defined by a single domain, while gradually fewer families are defined by multi-domain-sequences. Single-domain proteins also clearly outnumber the multi-domain proteins in the genomes, contrary to what is sometimes claimed ^35^. The longest domain sequence contains 25 non-overlapping Pfam-A hits in the same protein, mainly multiple copies of PF05662.9 and PF05658.9, both short repeats associated with haemaglutinins. This protein is found in 4 of the 347 genomes.

**Figure 3.**
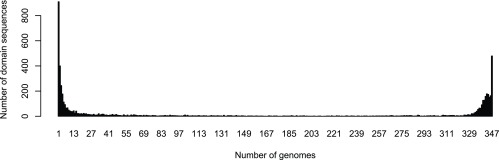
Gene family distribution. The distributions of how many domain sequence families are found in 1, 2, …, 347 genomes. There are 909 ORFan families (leftmost bar), 479 core families (rightmost bar) and in total there are 5724 unique domain sequence families (sum of all bars).

In Figure 3 we show the distribution (presence/absence) of domain sequences over the *E. coli* population. The leftmost bar is the number of ORFans, domain sequence families found in one single genome only. We found 909 ORFans when considering all 347 genomes. The rightmost bar are the 479 core domain sequence families found in at least one copy in all 347 genomes. The distribution is very similar in shape to what we usually see for pangenomes of other data sets and where gene families are computed. Hence, also the domain sequence families are distributed across genomes in this way.

**Figure 4.**
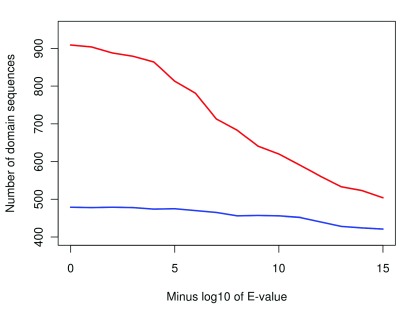
Effect of significance cutoff. The horizontal axis is the – log _10_ of the HMMER3 E-value cutoff and goes from *E* = 10 ^-0^ on the left to *E* = 10 ^-15^ on the right. The blue curve shows how the number of core families drops by stricter cutoff (going from left to right), and the red curve similar for the number of ORFan families.

The most remarkable result is the large number of ORFans. More than 15% of the domain sequence families found in *E. coli* are seen in only 1 out of 347 genomes. The Pfam-A models are curated and have a built-in threshold for assigning significant hits. However, given our approach, where we scan a list of presumably many false positive protein predictions, we may be extra restrictive when considering the hits. In Figure 4 we show how a systematic change in E-value cutoff changed the number of core families and ORFan families. As we lower the E-value cutoff, little happens to the number of core families, but the number of ORFans drops markedly. This demonstrates that the ORFan are, as usual, the most uncertain families. In the downstream analysis we made a parallel analysis using the E-value cutoff 10 ^-10^ as a strict alternative to the built-in thresholds.

Tabular overview of genomes An overview of the 347 genomes of Escherichia coli used in this study. This text file contains the BIOID code for each genome, the corresponding file name, short name (usually the strain) and whether the genome project is completed or not.

Archive of FASTA-formatted files containing predicted proteins for each genome A g-zipped archive with 347 text files containing the predicted proteins found by at least one of the gene finders Prodigal, Glimmer or GeneMarkHMM for each genome.

The 347 text files show the results from scanning each of the fasta-files against the Pfam-A database The 347 text files show the results from scanning each of the fasta-files against the Pfam-A database.

### Functional space

**Figure 5.**
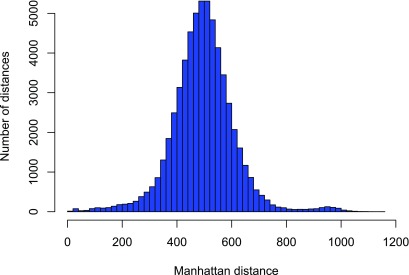
Functional distances. A histogram over all pairwise Manhattan distances between the genomes. The distance between two genomes is defined as the number of domain sequence families they differ in presence/absence status, i.e. a distance of 500 means there are 500 different families contained in one but not the other genome.

Domains are usually well conserved, and may show too few differences between strains within a species, i.e. all genomes contain more or less the same domain sequences. To examine this, we computed the Manhattan distance between every pair of genomes, which is simply the number of domain sequences where the two genomes differ in presence/absence status (ignoring copy numbers). Figure 5 shows a histogram of all pairwise distances. The median distance between two genomes is around 500, meaning there are 500 distinct domain sequences contained in one of the genomes but not the other. Only two genomes came out identical, and this is actually the same strain, BL21(DE3), sequenced at two different locations (Korea and Austria), but stored under unique accession numbers in the database. There are actually several other examples of multiple sequencing of the same strain in this dataset (2 versions of strain DH1, 2 of strain KO11FL, 3 of strain W), but in all other cases at least 3 or more differences are found between genomes. As an example, among the four K-12 strains in this dataset the differences in domain sequences are from 5 (between substrains MG1655 and W3110) to 94 (between substrains MG1655Star and DH10B). Hence, even very closely related strains of the same serotype have plenty of differences in domain sequence absence/presence. Using the strict E-value cutoff reduced the median distance to around 450, but did not change the shape of the histogram. The small 'bump' on the right hand side is due to a few genomes being quite different from the rest. These are all draft genomes with a large number of contigs, producing a smallish number of domain sequences.

We may think of each domain sequence as representing a small repertoire of functions the genome can inhabit. For each genome the vector of presence/absence of the set of domain sequences will correspond to a location in a functional space, and the Manhattan distance between two genomes in this space is a functional distance. A pangenome tree can be made from these Manhattan distances ^36^. However, any tree visualization will suffer when we have as many as 347 leaf nodes. Supplementary figure 1 illustrates a pangenome tree for the 347 *E. coli* genomes.

**Figure 6.**
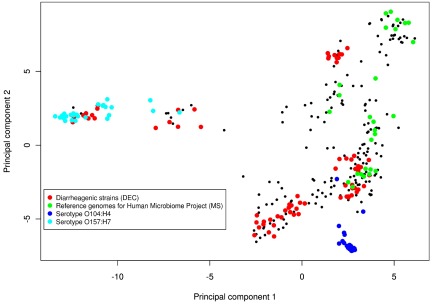
Genomes in functional space. Each dot corresponds to a genome plotted in the two first principal component directions of the *E. coli* functional space defined by the presence/absence of domain sequence families. There are four large subsets of genomes in the data set, and these dots are marked with colors, see figure legend. The first principal component accounts for 11% of the total data variation, and the second component 8%. Only relative positions of the genomes (dots) are important, the absolute scores on each axis lacks interpretation.

This sample of *E. coli* genomes contains several potential sub-samples, i.e. strains having more in common than just being of the same species. It is natural to see where these are located in this functional space, and here we have looked at the four largest sub-samples in the data set. In ^37^ it was found that ETEC type of strains shared some genomic features distinguishing them from other *E. coli*, and it is reasonable to expect that certain strain subsets will cluster within the total collection of *E. coli* genomes. We used a principal component analysis on the present/absent pan-matrix as described in the Methods section. This functional space is high- dimensional, i.e. many principal components directions are needed to explain the majority of the variation between genomes. In Figure 6 we have plotted each genome as a dot in the subspace spanned by first two principal components directions only. As seen from the axes labels these two directions account for less than 20% of the total variation between genomes, but still, the clustering of the enterohaemmoragic serotypes (O157:H7, light blue dots and 0104:H4, dark blue dots) is clearly visible. The collection of diarrheagenic strains (red dots) are clumped at several locations, indicating a mixture of functional content. The Human Microbiome Project strains (green dots) are all located to the right hand side of Figure 6. Using the strict E-value cutoff hardly changed the picture in Figure 6 at all. This is due to the extreme stability of a multivariate analysis like PCA. Since we know from Figure 4 that this strictness reduces the number of ORFans, it means the picture in Figure 6 is marginally influenced by ORFan gene families.

A pan-tree for 347 E. *coli* genomes The SVG-file is an unweighted  pan-tree for the 347 genomes of E. coli in this study.

Cluster information The file cluster_info.txt is a table with two columns. Each row corresponds to a domain sequence family. These are named Cluster 1, Cluster 2,...etc. In the second column is listed the ordered occurrence of Pfam-A domains for each domain sequence family.

Pan Matrix data The file pan_matrix.txt is a huge table (tab-separated columns) where each row corresponds to a genome and each column to a domain sequences family. The rows are named by the BIOID-code, see map_ecoli.txt to look up the strain names. The columns are named Cluster 1, Cluster 2,...etc. The corresponding Pfam-A domain sequence is given in the file cluster_info.txt (see below). In cell (i,j) in this table you find the number of occurrences that domain sequence j has in genome number i.

### Pangenome analysis

In this sample of 347 *E. coli* genomes we find 5724 unique domain sequences. Using the binomial mixture model suggested in ^14^, the total pangenome size is estimated to 6040. Employing the suggested bagging procedure, we find that in 90% of the re-sampled cases the estimate is between 5998 and 6136, reflecting the uncertainty in the data. This should be regarded as a lower bound estimate, but indicates we have already seen the majority of domain sequence families in *E. coli*. The observed set of domain sequences covers around 95% of the predicted pangenome.

We also conducted a Heaps law analysis as suggested by ^13^. From this it seems the population of *E. coli* domain sequences is open. We fitted the Heaps law model to these data, using 100 random permutations of genome ordering, and came up with the estimate 0.94 for the parameter α in the model. A value of α < 1.0 is consistent with an open population. This means the population size is unbounded when extrapolating the Heaps law trend into many more sequenced genomes. However, unbounded is a mathematical term, and an unbounded function can still grow very slowly. The model predicts that after 1 million sequenced *E. coli* genomes(!) we still have not seen more than 9442 domain sequences. As such, the result is not that different from the mixture model estimate.

We repeated the computations using the strict E-value cutoff in the HMMER3 software. The sample pangenome then reduces to 4745 (from 5724) observed domain sequence families, since only very significant ( *E* < 10 ^-10^) Pfam-A hits are now considered. The binomial mixture model gives a pangenome size estimate of 4973, which still means a coverage around 95%. The Heaps law analysis, however, results in a closed population, with α = 1.01 and an estimate of its size at 4876 domain sequences. This illustrates how the choice of cutoffs in the sequence clustering may change a result completely. The Heaps law analysis is extremely sensitive to the number of ORFans in the data set.

**Figure 7.**
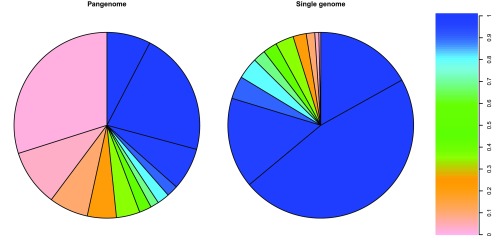
Binomial mixture model. The left pie chart visualizes the *E. coli* pangenome and the right pie chart a single *E. coli* genome described by a binomial mixture model. There are 12 sectors, and the colors indicate the selection probabilities as displayed on the right hand colorbar. The size of the each sector shows its relative contribution to the pangenome (left) or a single genome (right). The pangenome is dominated by domain sequences having either a very large (darker blue sectors) or very small selection probability (pink sectors). In a single genome the highly conserved domain sequences (darker blue) clearly dominate.

The sample core size is 479 domain sequences, and the binomial mixture model predicts the population core to be 462. However, the notion of the core is difficult, since we require a core domain sequence to be present in every single genome. Due to the uncertainties in gene prediction and computation of sequence clusters, such a crisp limitation of the core is unfortunate. From the binomial mixture model we get a much better and more robust picture by considering the estimated selection probabilities behind every mixture component. In Figure 7 we have visualized the *E. coli* pangenome as a pie chart, where the colors indicate the selection probabilities. Using the Bayesian Information Criterion (BIC) we found that 12 components was optimal for the current data set. This means *E. coli* domain sequences can be grouped into 12 distinct sectors with respect to how often they appear in the genomes. The core genes is one of these types, having selection probability 1.0 since they occur in every genome (darkest blue sector). Notice also the large sector of domain sequences with a selection probability of 0.988. Even the third darker blue sector has a selection probability of 0.966. The large number of domain sequence families in these sectors are also highly conserved, and could be seen as core families for all practical purposes.

The coloring in Figure 7 reflects the commonly used division of the pangenome into three types of genes; the core (dark blue), the shell (light blue/green) and the cloud (orange/pink) ^38, 39^. By allowing the mixture model to have many more components, we found 12 was optimal here, we can cope with the fact that genomes are not uniformly distributed within the population. The distribution in Figure 3 is affected by this, e.g. since we have 31 genomes of serotype O157:H7 in the sample we expect there will be a small ‘bump’ in the distribution at 31 genomes, reflecting the domain sequence families common to these closely related genomes. The binomial mixture approach can be illustrated by a lunch buffet table, with each single genome as a plate to be filled with content from the pangenome (the buffet table). Some families on the buffet are always selected, and turn up on every plate. These are the core families. The other families are more or less popular, and have different probabilities of being selected. Some families are very unlikely to be selected, but if there is a large number of them, some of them will end up in almost every genome. However, these ORFans are so unlikely to appear they are never seen twice.

The right hand pie in Figure 7 shows the expected distribution of domain sequence families within an average *E. coli* genome. Clearly, the darker blue domain sequences make up the majority. The size of the pangenome is largely due to the big number of ORFan domain sequences (pink sectors in left pie), but in any average genome these makes up a small minority (pink sectors in right pie). The expected overlap between two genomes is 0.90, i.e. on average 90% of the domain sequence families in one genome are also found in another. We also computed the genome fluidity to 0.11 and the mean Jaccard distance between two genomes to 0.18, both indicating a small uniqueness (lack-of-overlap) in each genome.

## Conclusion

Any study of pangenomes involves the clustering of sequences into gene families, and the results obtained will invariably depend on the way gene families are computed. In this paper we have proposed an alternative based on protein domains to obtain stable sequence clusters. The cost of this stability is the loss of some genes in the study, since only proteins with known domains are considered. We have used only the Pfam-A database, and by extending it to also include other databases (e.g. Pfam-B, CDD, InterPro), some more hits would probably be found, reducing this loss. Also, domain sequence families are large and 'coarse' compared to the protein families usually considered in pangenomics. However, the gain of this approach is a robustness with respect to gene prediction, eliminating many false positive proteins from the analysis as well as the potential effect of the inconsistent annotation of start codons. More attention should be given to improve gene prediction for prokaryotes, and the recent approach taken by Angiuoli *et al.* ^34^ is a good idea for pangenome data. The standard approach has been to predict genes in one genome at the time. When multiple strains are sequenced, we can instead find genes conserved across many genomes, and from this improve the consistency of start codon predictions, which is the major difficulty.

Another advantage of our proposed approach is the fact that Pfam-A domains have a built-in significance threshold optimized for each individual HMM, and by using this we obtain domain sequence families with variable heterogeneity reflecting the various degree of conservation of different types of proteins. The procedure for computing such domain sequence based gene families is straightforward and highly reproducible, using only publicly available software. Finally, each genome is only scanned once, which means the computational load scales linearly in the number of genomes.

Despite that domain sequence families are large, with potential low resolution between strains, we find plenty of differences in *E. coli* strains. We also find meaningful groupings of strains, indicating that the differences are not just random 'noise' but have plenty of biological foundation. The use of domain sequences for classification of strains clearly has some potential, and this should be pursued further.

With as many as 347 genomes, and more than 1000 soon available, we would expect the *E. coli* pangenome to be fairly well covered. By counting domain sequence families, this seems to be the case. Our lower bound estimate of pangenome size indicates we have seen perhaps as much as 95% of all domain sequences ever to be found in this species. Domain databases, like Pfam-A, will still grow but the number of single domain proteins is leveling out, and the future growth will mainly be due to new domain sequence combinations ^40^. There is an endless number of possible domain combinations even with todays databases, and for *E. coli* we must expect some new combinations in future sequenced genomes. However, with an overlap of 90% between any two *E. coli* genomes the time of big surprises is gone. The Heaps law analysis indicates the population is open, but we believe this analysis is too sensitive to a smallish number of ORFan families in the data set. Also, the entire concept seems to build on the assumption of a uniform sampling of strains, which is clearly not the case for *E. coli*.
