# Supplementary material for: A domain sequence approach to pangenomics: applications to Escherichia coli
Source: F1000Res. 2013 May 29;1:19. Originally published 2012 Oct 1. [Version 2] doi: 10.12688/f1000research.1-19.v2 (PMC3901455; doi:10.12688/f1000research.1-19.v2)
Supplement: Peer review history

# Peer review: Referee response for version 2

**Published:** 2013-05-30

**Authors:** Frederic Bertels

**Affiliations:** 1 Biozentrum, University of Basel, and Swiss Institute of Bioinformatics, Basel, Switzerland

The authors present a very nice study and I think the revisions have made this paper even better and clearer.

I have read this submission. I believe that I have an appropriate level of expertise to confirm that it is of an acceptable scientific standard.

# Peer review: Referee response for version 1

**Published:** 2012-10-15

**Authors:** Barry Wanner

**Affiliations:** 1 Department of Molecular Biosciences, Purdue University, West Lafayette, IN, USA

The authors provide an excellent-outstanding computational analyses of the *e. coli* pangenome with respect to protein coding across more than 50 complete genomes and 350 complete and whole genome shotgun (incomplete) genomes.

Importantly, they evaluate the proteins and protein families by recomputing/predicting families, rather than relying on GenBank/RefSeq annotations, which can vary depending upon source. Their work is well-documented and should serve as a gold standard for pangenome analyses of protein families for many bacteria.

There are however, some minor improvements that can be made to this paper:

**1.** On top right column of page 7, the authors explicitly state that “Only two genomes are identical… BL21(DE3). Since several non-identical K-12 strains are included in their Data File(MG1655, W3110, DH10A, and a couple others), authors should simply state how many K-12 strains were included in their analyses.

**2.** It would be especially helpful to the community, if the authors can post data files of their analyses, e.g., the raw data used to generate their pie charts in Figure 7. I am sure users would more widely cite their efforts if such results can be made easily accessible.

I have read this submission. I believe that I have an appropriate level of expertise to confirm that it is of an acceptable scientific standard.

# Peer review: Referee response for version 1

**Published:** 2012-10-04

**Authors:** Frederic Bertels

**Affiliations:** 1 Biozentrum, University of Basel, and Swiss Institute of Bioinformatics, Basel, Switzerland

The authors provide a very interesting insight into how one can reliably identify genes and classify them into gene families in fully sequenced bacterial genomes and apply this approach to 347 fully sequenced *Escherichia coli *genomes.

They avoid annotation problems by first running a very crude gene prediction program, that returns a large number of genes with a large proportion of false positive open reading frames (ORFs). For these ORFs they then predict Pfam-A domains. All ORFs for which no domain could be predicted were discarded. They then show that the remaining set open reading frames closely resembles the number of ORFs expected from manually curated genome annotations. They then go on and analyse the domain sequence composition of all remaining ORFs and provide a comprehensive pan genome analysis. In conclusion, the paper presents a very interesting approach to solve gene classification problems in pan-genome studies, a field that rapidly gains significance as whole genome sequencing becomes cheaper and cheaper.

The paper is well written and very comprehensive. The data is presented clearly and is directly downloadable from within the paper. The author even explain complex problems with interesting similes, which I thought was a nice idea and helped me to understand the presented ideas.

There are only a few minor problems that should be addressed:

**1.** One thing needs clarifying. The authors mention in paragraph two on page five that within the RefSeq annotated genomes they can identify 2725 of the 2750 unique domain sequence families for genes that were automatically identified. Also they say that they identify 100 extra unique domain sequence families in automatically identified genes.Does that mean that on average 2825 unique domain sequence families were identified? If this is so then I do not understand why in the second to last paragraph the authors write that in every single genome there are about 2500 unique sequence domain sequences. Especially since Figure 1 shows that on average there are more proteins with Pfam hits for draft genomes, and draft genomes are on average longer. Does that have to do with the fragmentation of the data that leads to splitting of domains? If so, how would that affect functional predictions?

**2.** How do the authors deal with gene predictions where one gene is predicted on the top strand and another on the bottom strand or generally genes predicted in different reading frames by different gene prediction programs? Did the authors test whether the data that was missed by/additional to the refseq annotation were overlapping genes but in different reading frames and hence could not have been picked up by Pfam no matter what the threshold is?

**3.** I think it would be interesting to estimate the effect that errors in gene prediction have on prediction of metabolic function etc. would have in theory. For example, what were the annotations of the unique domain sequence families that were missed by refseq or the gene finders. Were those mostly proteins of unknown function or did it include proteins which could potentially be predictive for the environment a particular bacterium could survive in?

**4**. The paper does need some proof reading, there are quite a few grammatical mistakes.

**5.** Throughout the paper the authors confuse the word “that” or “which” with “who”. For example in the abstract it says: “Clustering sequences by their ordered domain content give us domain sequence families, who are robust to errors in the gene prediction step.” In this case “who” refers to “domain sequence families” and hence should be substituted with “that” or “which”.

**6.** The authors frequently use the words “eukaryote” and “prokaryote” as adjectives (e.g. third paragraph of the introduction: “Even if prokaryote genes are in most cases simpler to detect than eukaryote counterparts, there are still problematic cases.”) The proper adjective should be “eukaryotic” or “prokaryotic” (and in the example it should probably also say “their eukaryotic counterparts”).

**7.** On page four, in the Methods part of the paper, the authors present a formula for the binomial mixture model. In this formula the running variable is k but changes to kappa in a few places (in the formula as well as in the text). This is a bit confusing and needs fixing.

**8.** In a few places references are given in the following format: “as described in ^36^” in those cases either the “as described in” part should be left out or it should say as described by Snipen and Ussery ^36^″ or “as described in one of our earlier papers ^36^“.

**9.** There are quite a few cases where plural and singular are confused, for example in the last paragraph of page 7 it says “in the first two principal component” it should say “first two principal components”.This particular sentence is also a bit confusing and may need rephrasing. There are other cases where the authors confused “was” and “were”, for example in the last paragraph on page 9 it says “Using the Bayesian Information Criterion (BIC) we found that 12 components was optimal for the current data set.” It should either say “dividing the data set into 12 components was optimal” or “12 components were optimal”.

**10.** The figure caption of Figure 7 requires some revision.

**11.** In the first paragraph of page 11 the reference for Angiouli *et al.* is missing. The authors also mention in this sentence the recent approach of Angioula et al without explaining what this approach is and how it compares to the presented approach. After reading the manuscript again I noticed that this paper was mentioned also in the beginning of the Results and Discussion section, but I still think it would be helpful for the reader if the authors briefly outlined what Angiouli *et al* did to improve consistency and in particular how this could be integrated into the presented approach.

**12**. In general the Conclusions section is harder to read and less clear than the rest of the paper, maybe it would help to spend a little bit of time revising this section.

I have read this submission. I believe that I have an appropriate level of expertise to confirm that it is of an acceptable scientific standard.
